# Antigenotoxic Effects of Biochaga and Dihydroquercetin (Taxifolin) on H_2_O_2_-Induced DNA Damage in Human Whole Blood Cells

**DOI:** 10.1155/2019/5039372

**Published:** 2019-11-11

**Authors:** Lada Živković, Vladan Bajić, Dijana Topalović, Marija Bruić, Biljana Spremo-Potparević

**Affiliations:** ^1^Center for Biological Research, Faculty of Pharmacy, University of Belgrade, Vojvode Stepe 450, 11000 Belgrade, Serbia; ^2^Laboratory for Radiobiology and Molecular Genetics, Institute for Nuclear Research “Vinča”, University of Belgrade, Mike Petrovića Alasa 12-14, 11000 Belgrade, Serbia

## Abstract

The health benefits of natural products have long been recognized. Consumption of dietary compounds such as supplements provides an alternative source of natural products to those obtained from the diet. There is a growing concern regarding the possible side effects of using different food supplements simultaneously, since their possible interactions are less known. For the first time, we have tested genotoxic and antigenotoxic effects of Biochaga, in combination with dihydroquercetin. No genotoxic effect on whole blood cells was observed within individual treatment of Biochaga (250 *μ*g/mL, 500 *μ*g/mL and 1000 *μ*g/mL) and dihydroquercetin (100 *μ*g/mL, 250 *μ*g/mL and 500 *μ*g/mL), nor in combination. Afterwards, antigenotoxic potency of both supplements against hydrogen peroxide- (H_2_O_2_-) induced DNA damage to whole blood cells (WBC) was assessed, using the comet assay. Biochaga and dihydroquercetin displayed a strong potential to attenuate H_2_O_2_-induced damage on DNA in cells at all tested concentrations, with a statistical significance (*p* < 0.05), whereas Biochaga at the dose of 500 *μ*g/mL in combination with dihydroquercetin 500 *μ*g/mL was most prominent. Biochaga in combination with dihydroquercetin is able to protect genomic material from oxidative damage induced by hydrogen peroxide *in vitro*.

## 1. Introduction

The health benefits of natural products have long been recognized. Regular consumption of dietary polyphenols is associated with reduced risk of a number of chronic diseases, including cancer, cardiovascular disease, and neurodegenerative disorders [1], suggesting that polyphenol compounds are able to neutralize harmful oxidative effects of reactive oxygen species (ROS). Because of their antioxidant qualities, polyphenols have received increased attention. Flavonoids are classified as a family of polyphenol compounds that are ubiquitous in nature and are consumed as part of a human diet in significant amounts [2].

Taxifolin (2R,3R-dihydroquercetin) is a dihydroflavonol, a bioactive component that is a part of the Siberian larch, *Larix sibirica* Ledeb., native to Russia. Taxifolin can be found in other conifers such as the Chinese yew, *Taxus chinensis* var. *mairei* (Lemee et Levl) Cheng et L.K. Fu, but also in the conifers from the Mediterranean area [3]. There is evidence that taxifolin protects bmMSCs from ·OH-induced damage [4] and inhibits free radical formation at key stages of apoptosis in cellular mitochondria [5]. It was reported that taxifolin displayed anticancer and neuroprotective properties [6–8].

Chaga (*Inonotus obliquus* (Ach. exPers.) Pilat) is a parasitic Polyporus from the Hymenochetaceae family. Chaga grows primarily in Russia, but also in parts of Japan, Korea, Alaska, Canada, and northern Scandinavia. This fungus infects hardwood trees, mostly those from the genus *Betula* (birches), and to a lesser extent, those from the genera *Quercus* (oaks), *Populus* (poplars), *Alnus* (alders), *Fagus* (ashes), and *Acer* (maples) [9]. Chaga has been used since the 12th century in Siberia, as an edible medicinal mushroom for the prevention and treatment of cancer, as an antitubercular, to cure digestive disorders, or to prevent cardiac or hepatic illnesses [10]. Its bioactive components exhibit antitumor, anti-inflammatory, hypoglycemic, and immunomodulatory effects, as well as antioxidative properties [11]. Regarding the chemical analyses of Chaga, the polyphenols, triterpens, and polysaccharides could be liable for its therapeutic properties [12].

In aerobic cells, ROS are continuously derived as a byproduct of normal mitochondrial activity or by external sources. If not properly controlled, ROS can cause severe damage to cellular macromolecules, especially to DNA [13]. Multiple DNA modifications such as base damage, sugar damage, and DNA protein crosslinks resulting in single-strand and double-strands breaks are generated by hydrogen peroxide (H_2_O_2_). Such extent of the DNA damage can determine the fate of a cell: cell cycle arrest and DNA repair or the activation of apoptotic pathways or promotion of tumorigenesis as well as initiation of immune response and inflammation [14].

Consumption of dietary supplements provides an alternative source of bioactive natural compounds to those obtained from the diet. There is a growing concern regarding possible side effects of using different flavonoid-containing food additives simultaneously, since possible flavonoid combination effects are less known [15]. The safety assessment is required.

Our work is one of the first experimental approaches on Chaga collected in Siberia and dihydroquercetin extracted from Siberian larch. This contribution is aimed, on the one hand, at evaluating the antigenotoxic effects of Chaga, as well dihydroquercetin extracts against H_2_O_2_-induced DNA damage on whole blood cells (WBC) *in vitro,* and on the other hand, to test its possible synergistic effects against oxidative DNA damage. To address our objectives, the protective potential of analyzed compounds was estimated by using the comet assay, a well-established, highly sensitive method for examining DNA damage within cells.

## 2. Material and Methods

### 2.1. Subjects

Peripheral blood samples were collected from four female and two male subjects, between the age of 20 and 25 years. The subjects did not consume medicaments or food supplements. They gave their consent in accordance with the regulations of the ethical standards of Ethics Committee for Clinical Trials of the Faculty of Pharmacy, University of Belgrade.

### 2.2. Methods

Commercial products of Biochaga and dihydroquercetin capsules (Sibpribor, Irkutsk, Russia) were tested. The powder was dissolved in phosphate-buffered saline (PBS, Fisher Scientific, Pittsburgh, PA), stirred for 30 min at 37°C, and filtered through a filter paper. Concentrations of 250 *μ*g/mL, 500 *μ*g/mL, and 1000 *μ*g/mL of Biochaga and 100 *μ*g/mL, 250 *μ*g/mL, and 500 *μ*g/mL of dihydroquercetin were used to perform the comet assay analysis. The concentrations of tested substances were chosen according to literature data [4, 16] and corresponded to the ones used in our previous studies of medicinal mushrooms [17, 18].

#### 2.2.1. Evaluation of Genotoxic and Antigenotoxic Effects of Biochaga and Dihydroquercetin by Comet Assay

To estimate genotoxic and antigenotoxic properties of the tested products, the comet assay was conducted. The following treatments were performed: (a) to evaluate the genotoxic effect, the cells were treated separately with Biochaga and dihydroquercetin in tested concentrations at 37°C for 30 min, where for the positive control we used H_2_O_2_ (ZORKA Pharma, Šabac, Serbia) exposure; (b) in order to estimate the antigenotoxic effects of the tested compounds, cotreatment was conducted, i.e., cells were simultaneously exposed to H_2_O_2_ and the tested supplements for 30 min at 37°C, and cotreatment with quercetin 100 *μ*g/mL, 250 *μ*g/mL, and 500 *μ*g/mL was used as the positive control; (c) to assess possible benefits or side effects of Biochaga and dihydroquercetin interactions, we simultaneously treated cells with both products and applied both experimental designs (genotoxic and antigenotoxic assessment). A concentration of 50 *μ*M H_2_O_2_ was used, since it was the lowest concentration that induced statistically significant increase of level of DNA damage in human peripheral whole blood cells.

#### 2.2.2. Kinetics of Attenuation of H_2_O_2_-Induced DNA Damage with Biochaga and Dihydroquercetin Treatment by Comet Assay

To estimate the kinetics of attenuation of H_2_O_2_-induced DNA damage in WBC in the posttreatment group with Biochaga and dihydroquercetin, the most effective concentrations were chosen from prior antigenotoxic assessment. Following 30 min of H_2_O_2_ at 4°C, cells had been treated separately with the Biochaga and dihydroquercetin at 37°C for 4 time periods: 15, 30, 45, and 60 min.

#### 2.2.3. The Single-Cell Gel Electrophoresis (Comet Assay)

The comet assay was performed as described by Singh and colleagues [19]. Prior the treatments, samples of peripheral blood (6 *μ*L) were suspended in 0.67% of low-melting-point agarose (Sigma-Aldrich, St. Louis) and pipetted onto superfrosted glass microscope slides precoated with a layer of 1% normal-melting-point agarose (Sigma-Aldrich, St. Louis, MO), spread by a coverslip, and maintained for 5 min in the freezer to solidify. Aiming to evaluate genotoxic and antigenotoxic effects of tested supplements, the coverslips were removed and the samples were treated according to two experimental conditions. Following the treatment, samples were covered with a layer of 0.5% low-melting-point agarose and recooled for 5 minutes at 4°C. When the coverslips had been removed, the slides were placed in a precooled lysing solution (2.5 M NaCl, 100 mM EDTA, 10 mM Tris, 1% Triton X100, and 10% dimethylsulfoxide, pH 10 adjusted with NaOH) for 24 hours at 4°C. The next day, the samples were subjected to electrophoresis. Slides were placed in the horizontal gel electrophoresis tank and overflooded with cold, fresh electrophoresis buffer (10 M NaOH, 200 mM EDTA), which allows DNA to denature before electrophoresis. After 30 min in fresh the electrophoresis buffer, electrophoresis was performed under dimmed light at 25 V and 300 mA. Defined pH values and the strength of the electric field make DNA mobile and cause the distance that the fragments pass will depend on the degree of damage of the DNA molecule. After electrophoresis, the samples were washed twice with neutralizing buffer and once with distilled water, with a time interval of 10 minutes between washings.

Finally, the samples were stained with 50 *μ*L ethidium bromide (20 mg/mL) and after 15 minutes observed under Olympus BX50 microscope (Olympus Optical Co. GmbH, Hamburg, Germany), equipped with a mercury lamp HBO (50 W, 516-560 nm, Zeiss) pigment lens and at magnification of 100x.

#### 2.2.4. Assessment of the Degree of DNA Damage

All samples were made in duplicate, and on each slide 100 randomly selected cells were counted. An impact of the electric field make the negatively charged fragments of DNA molecules migrate towards the anode by making a comet tail, while the rest represents the head of the comet. DNA damage was estimated according to Anderson and colleagues [20]. Depending on the degree of damage of DNA molecules, the comets are classified into five categories: category A (no damage, <5%), category B (low level of damage, 5-20%), category C (mean damage level, 20-40%), category D (high damage level, 40-95%), and category E (total damage, >95%).

### 2.3. Statistical Analysis

The statistical analysis was performed using analysis of variance (one-way ANOVA), with Tukey's post hoc or Student's *t* test for comparisons of different treatments versus the respective controls. Data were expressed as mean ± standard error of the mean (SEM), with *n* = 6. A difference at *p* < 0.05 was considered statistically significant. GraphPad Prism (6.0) statistical software (GraphPad Software Inc., La Jolla, CA, USA) was used for the analysis.

## 3. Results

### 3.1. Genotoxic Properties

**Figure 1:**
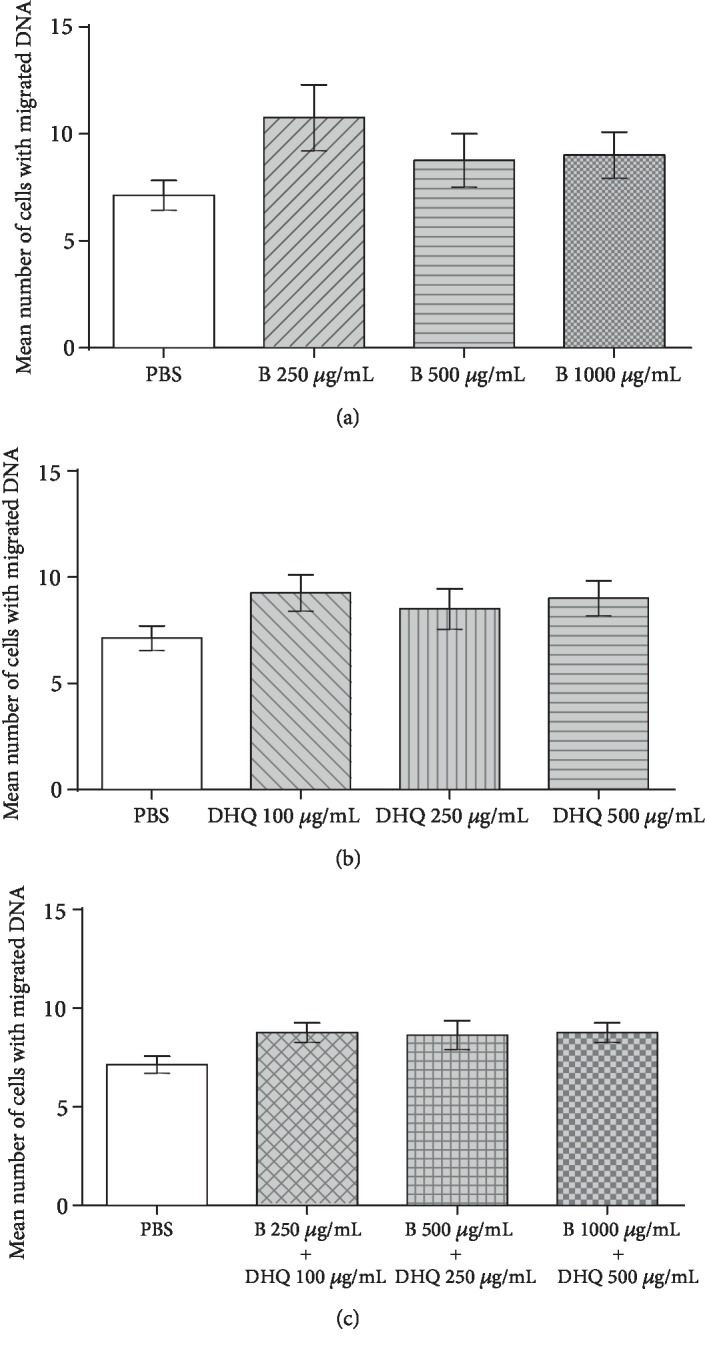
The evaluation of the genotoxic effects of (a) Biochaga at 250 *μ*g/mL, 500 *μ*g/mL, and 1000 *μ*g/mL after 30 min of incubation at 37°C, (b) dihydroquercetin at 100 *μ*g/mL, 250 *μ*g/mL, and 500 *μ*g/mL after 30 min of incubation at 37°C, and (c) simultaneous treatment of Biochaga and dihydroquercetin after 30 min of incubation at 37°C. Bars represent mean number of cells with DNA damage ± SEM, for *n* = 6 (by one-way ANOVA).

In the evaluation of the genotoxic potential of Biochaga, as well as dihydroquercetin, it was found that the tested range of concentrations applied to agarose-embedded blood cells did not increase the number of cells with DNA damage in comparison to the control (i.e., the PBS), for both of the separately tested compounds (Figures 1(a) and 1(b)). The simultaneous treatment of Biochaga and dihydroquercetin in all the tested range of concentrations indicated no genotoxic effects (Figure 1(c)).

### 3.2. Antigenotoxic Properties

**Figure 2:**
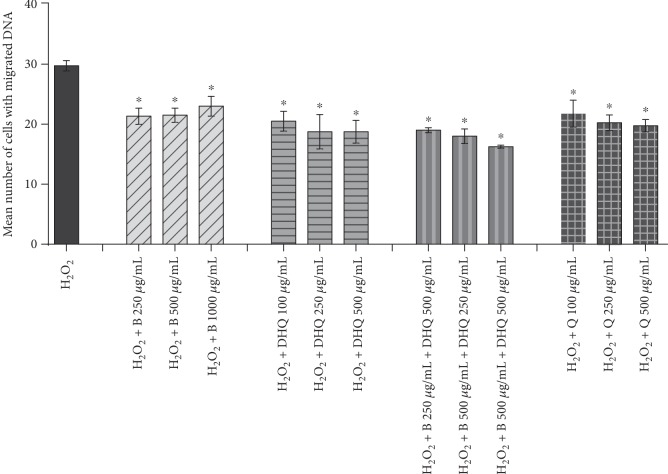
The evaluation of antigenotoxic properties of separate and simultaneous application of Biochaga and dihydroquercetin in the range of tested concentrations against H_2_O_2_-induced DNA damage in cotreatment. Quercetin (Q) in tested concentrations was used as a positive control. Bars represent mean number of cells with DNA damage ± SEM, for *n* = 6. ^∗^*p* < 0.05 vs. H_2_O_2_-treated cells (by one-way ANOVA).

In order to evaluate the antigenotoxic potential of Biochaga and dihydroquercetin, cotreatment with H_2_O_2_ and tested products was conducted on the peripheral blood cells. Our data revealed that Biochaga, as well as dihydroquercetin, in separate treatments, significantly decrease the number of cells with H_2_O_2_-induced DNA damage in the range of all the tested concentrations. Interestingly, the concentration of 250 *μ*g/mL displayed the most prominent reduction of DNA damage vs. control, in both cotreatments (Biochaga and dihydroquercetin). It should be mentioned that the concentration of 500 *μ*g/mL, for both products, displayed a similar level of attenuation of DNA-damaged cells regarding the most prominent concentration of 250 *μ*g/mL (Figure 2). Next, the cells were treated with a combination of Biochaga and dihydroquercetin in the concentration range of 250 *μ*g/mL and 500 *μ*g/mL, respectively. The cells were cotreated with H_2_O_2_. Our results showed (Figure 2) a significant reduction of cells with DNA damage in all tested groups vs. control. The simultaneous treatment with Biochaga 500 *μ*g/mL and dihydroquercetin 500 *μ*g/mL was the most efficient in the reduction of the number of damaged cells.

**Figure 3:**
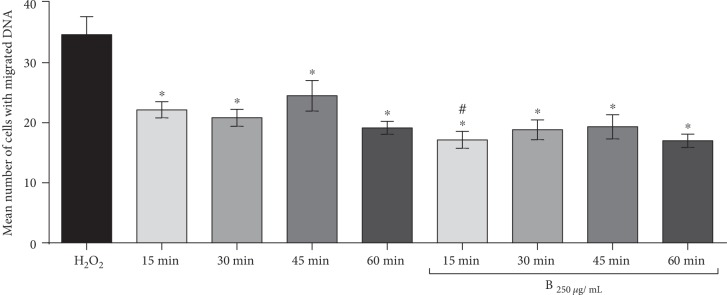
The time course of attenuation of DNA damage exposed to H_2_O_2_ and afterwards incubated for 15, 30, 45, and 60 min (a) without any treatment and (b) with 250 *μ*g/mL of the Biochaga. Bars represent mean number of cells with DNA damage ± SEM, for *n* = 6. ^∗^*p* < 0.05 vs. H_2_O_2_-treated cells; ^#^*p* < 0.05 vs. 15 min without any treatment (by one-way ANOVA and Student's *t* test).

**Figure 4:**
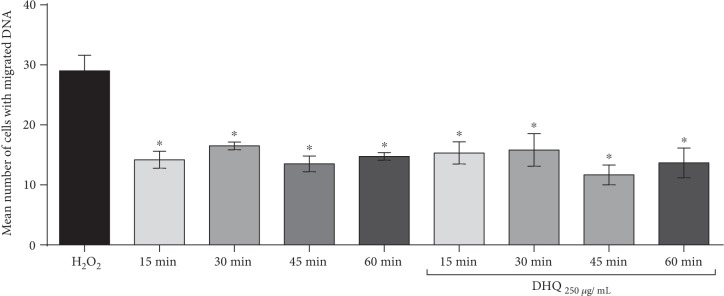
The time course of attenuation of DNA damage exposed to H_2_O_2_ and afterwards incubated for 15, 30, 45, and 60 min (a) without any treatment and (b) with 250 *μ*g/mL of the dihydroquercetin. Bars represent mean number of cells with DNA damage ± SEM, for *n* = 6. ^∗^*p* < 0.05 vs. H_2_O_2_-treated cells (by one-way ANOVA).

Regarding the results of separate cotreatments of tested compounds and H_2_O_2_, the concentrations of 250 *μ*g/mL for Biochaga, as well as for dihydroquercetin, were chosen as the most efficient in their action on the intervention level. Therefore, we have investigated a time course of attenuation of H_2_O_2_-induced DNA damage cells in a separate applied posttreatment experiment with Biochaga and dihydroquercetin, in order to better understand possible individual mechanisms of action of two different supplements. The posttreatment with Biochaga of cells previously exposed to H_2_O_2_ showed significant attenuation of damaged cells in all estimated time points. Similarly, in the control group, cells without additional treatment displayed a potential to attenuate the level of H_2_O_2_-induced DNA damage. It should be emphasized that Biochaga at 15 min after the H_2_O_2_ exposure significantly decreased the number of DNA-damaged cells in comparison to the same time point in the control group (Figure 3).  Figure 4 represents results of the attenuation of H_2_O_2_-induced DNA damage of cells in 4 time periods of posttreatment: 15, 30, 45, and 60 min, both in posttreated cells with dihydroquercetin and control (the cells were exposed to PBS and examined at the same intervals). In the presence of dihydroquercetin at time points 15, 30, 45, and 60 min, there was significant attenuation of H_2_O_2_-induced damage in all tested time points, but the control group also displayed significant attenuation of damaged cells in the tested time course. Our data revealed that the greatest reduction has been observed at 45 min after the exposure of the H_2_O_2_-treated cells to dihydroquercetin (Figure 4).

## 4. Discussion

The DNA is a most sensitive molecule where damage can occur through exogenous and endogenous ROS processes. DNA damage is regarded as the primary cause of cancer. In order to assess genotoxic and antigenotoxic properties of Biochaga and dihydroquercetin in individual human whole blood cells, DNA strand breaks were measured by using a sensitive and reproducible technique, the comet assay.

Regarding the findings of Ames and Gold [21] that numerous foods contain natural chemicals that damage DNA and might produce much greater impact on the genomic material than DNA damage produced by industrial chemicals, the investigation of the genotoxic effects of natural compounds, especially assessment of possible side effects of using simultaneously different contents of food additives, is needed. No genotoxic effect was detected by using the comet assay with regard to separate and simultaneous treatments with Biochaga and dihydroquercetin on human whole blood cells.

Hydrogen peroxide, together with various reactive oxygen species, is generated as a normal product of cellular metabolism, but excess hydrogen peroxide is one of the major contributors to oxidative DNA damage and might be considered to be the primary cause of spontaneous mutations and possibly have a role in further tumor genesis [22–24].

Cotreatment was conducted in order to estimate the tested compounds on the interventional level, via whole blood cell treatment with three concentrations for 30 min during the administration of the oxidant. The statistically significant efficiency of Biochaga to attenuate H_2_O_2_-induced DNA damage in all tested concentrations was detected. It was shown by Park and colleagues [16] that administration of Chaga in pretreatment, on prevention level, was protective against oxidative damage to DNA in human lymphocytes. Also, Chaga extract reduces oxidative stress in lymphocytes from inflammatory bowel disease patients and controls when challenged *in vitro* [25]. Similarly, all tested concentrations of dihydroquercetin were statistically significant in decreasing the number of DNA-damaged cells induced by H_2_O_2_. It has been reported by Manigandan and colleagues [26] that taxifolin (dihydroquercetin) exhibited a strong protection against OH-mediated DNA damage on pUC19 plasmid DNA. Interestingly, a simultaneous application of both products displayed a stronger potential to decrease the number of cells with H_2_O_2_-induced DNA damage vs. individual cotreatment with H_2_O_2_.

Quercetin effectively reduced H_2_O_2_-induced DNA damage in cotreatment, implying that the ROS attack on DNA underlies the origin of strand breaks. Our data indicates that Biochaga and dihydroquercetin also have the ability to attenuate free radical-induced DNA strand breaks.

A significant antigenotoxic effect was determined on the interventional level in the cotreatment experiment. This could be explained by an additional activation of independent mechanisms for making the cell genome more resistant to oxidative damage in the presence of tested compounds: free radical scavenging, enhancement of the cell antioxidant capacity, and stimulation of cell's DNA repair [27].

Considering the previous findings that potent cell's repair of DNA damage occurs within 1 h after the exposure to the oxidative agent [28, 29], we investigated the kinetics of attenuation of H_2_O_2_-induced DNA damage in the time period of 1 h. In cells which were treated with the appropriate concentration of Biochaga, as well as dihydroquercetin, and compared with untreated cells (exposed only to PBS), the results showed that separate posttreatments with both products significantly decreased the level of DNA damage in cells in 15, 30, 45, and 60 min time periods vs. H_2_O_2_ treatment, while the cells treated only with PBS also displayed significant reduction of the DNA damage in the same time frame but with lower attenuation capacity. It should be noted that the greatest reduction was observed 15 min after the exposure of the cells to Biochaga, with a statistical significant decrease of DNA-damaged cells in comparison to the same time point in the control group. The possible explanation of such prominent Biochaga effect at the 15 min time point could be its strong rapid scavenger ability. The study of Ham and colleagues [30] showed a strong antioxidant activity of Chaga against DPPH radicals. Significant radical scavengers from the methanolic extract of Chaga showed to act against ABTS and DPPH radicals [31].

Otherwise, the results of control groups which confirmed that the cell's repair capacity is activated in the presence of an oxidant and significantly contributes to attenuation of oxidative DNA damage are in support of the findings of Benhusein and colleagues [28] and Chiaramonte and colleagues [29]. On the other hand, Biochaga and dihydroquercetin displayed a potential to additionally stimulate cell's repair, but without any statistical significance vs. control groups. Namely, it has been established that mushrooms can demonstrate their antioxidant effects at different levels of the oxidation process owing to various mechanisms [32]. *Ganoderma japonicum* has potential therapeutic use as a DNA repair stimulator [33]. The study of da Silva and colleagues [34] showed the protective effect of *β*-glucan extracted from *Agaricus blazei* on the expression of the gene ERCC5 (involved in excision repair of DNA damage) on HepG2 cells. Also, Ramos and colleagues [35] have shown that flavonoids increased the rate of DNA repair in t-BHP-induced DNA damage in the human hepatoma cell line (HepG2).

The accumulation of oxidative DNA lesions predisposes individuals to accelerated tissue aging, neurodegeneration, and cancer [36–39]. Antioxidant-rich nutritional supplementation might hold a good strategy to attenuate oxidative stress and prevent against the related disorders. The results of the current study and previous findings regarding Chaga [16] and dihydroquercetin [40] propose a use of Chaga and dihydroquercetin as a dietary supplement to ameliorate oxidative instability and oxidative stress-related disorders. The current study excludes hazards of simultaneous application of tested supplements and promotes the evidence of their antigenotoxic properties against free radical damage to genetic material. *In vitro* testing provides capacity for further investigations in preclinical and clinical assays to determine their safety and antigenotoxic potential [41].

## 5. Conclusion

Current study shows antigenotoxic properties of Biochaga, as well as dihydroquercetin on human peripheral blood cells against oxidative effect of H_2_O_2_ in DNA through an interventional level. Also, for the first time, it was shown that Biochaga in combination with dihydroquercetin is able to protect genomic material from oxidative damage. Mechanisms underlying the antigenotoxic effects of investigated supplements should be further evaluated in *in vivo* studies.

## Data Availability

The data used to support the findings of this study are available from the corresponding author upon request.
